# Supplementary material for: Allelic variation of TaWD40-4B.1 contributes to drought tolerance by modulating catalase activity in wheat
Source: Nat Commun. 2023 Mar 2;14:1200. doi: 10.1038/s41467-023-36901-6 (PMC9981739; doi:10.1038/s41467-023-36901-6)
Supplement: Supplementary file 1 — Supplementary Information [file 41467_2023_36901_MOESM1_ESM.pdf]

Allelic variation of *TaWD40-4B.1* contributes to drought tolerance by modulating catalase activity in wheat

Tian *et al*.

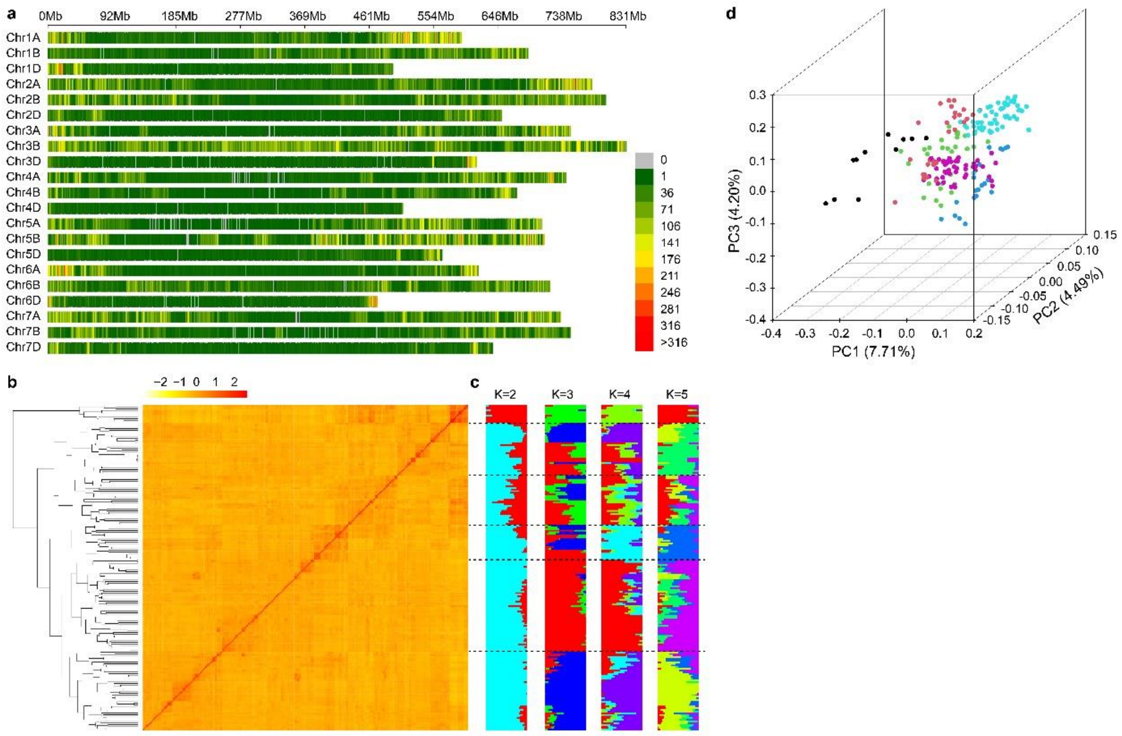

Supplementary Figure 1. The genetic structure of selected wheat accessions. a: The distribution of filtered SNPs across the wheat genome. **b**, **c**: Population structure revealed by kinship (**b**) and structure (**c**) analyses. **d**: The scatter plot of the first three principal components. The dots with different colors indicate the groups in panel b.

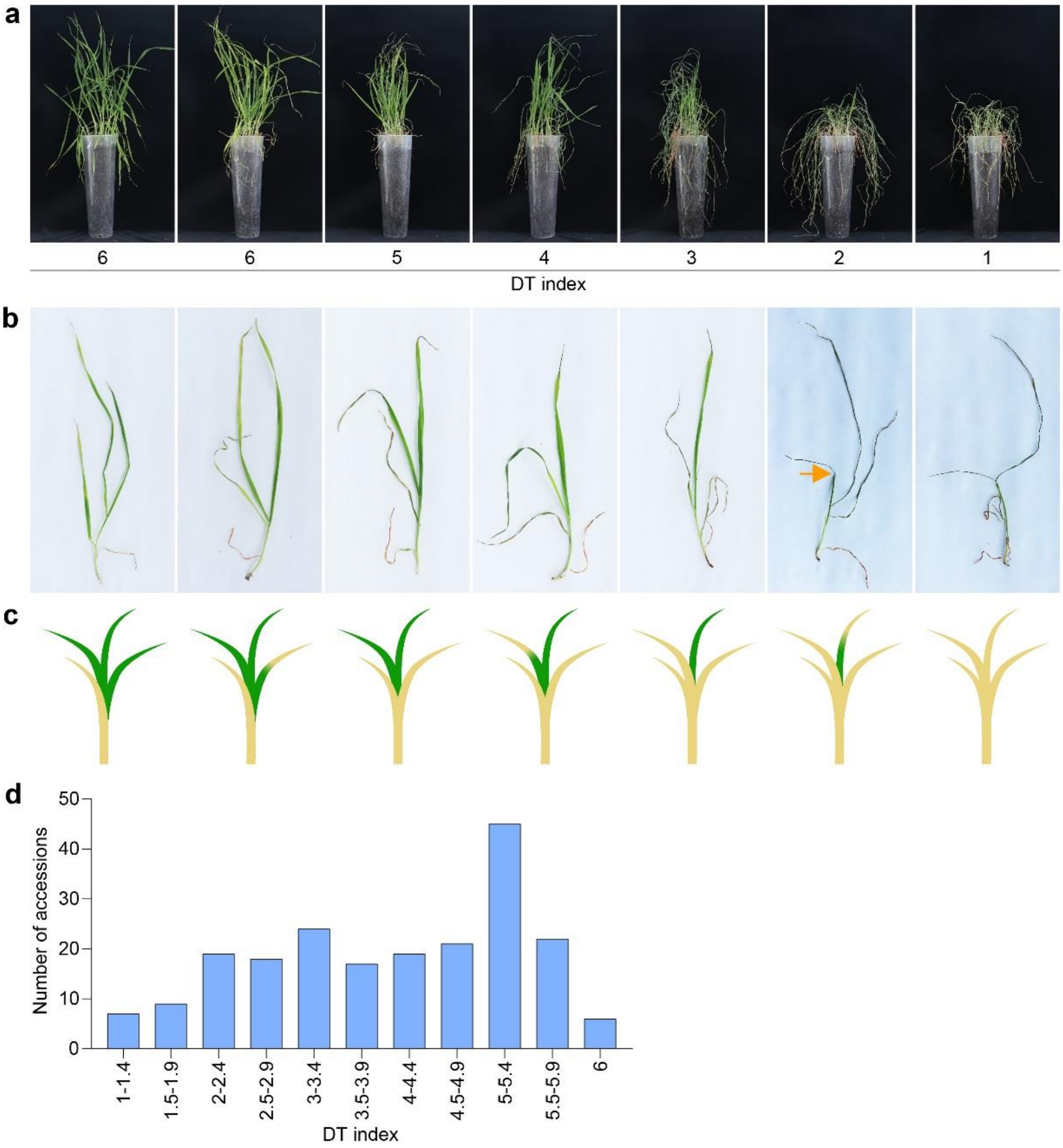

Supplementary Figure 2. The phenotype assay of the selected wheat accessions. a: The classification of wilting indices and drought tolerance (DT) indices. **b**: The representative seedlings with different wilting indices and DT indices. **c**: The cartoon diagram shows the seedlings with different wilting indices and DT indices. **d**: The distribution of DT indices of wheat accessions.

a

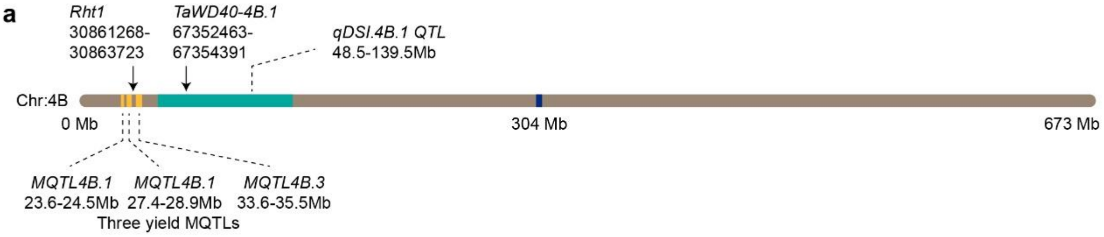

b

	Trait	Population	Refs
Bread wheat	qDSI.4B.1 QTL (including MQTL4B.1;4B.2;4B.3; Rht1)	NILs	1
	Leaf dry biomass	GWAS	2
	Plant height (18.24–21.59 cM), RDM (21.59–29.11 cM)	DH population	3
	Grain yield, TGW	Meta-QTL	4
	Plant height	RIL population	5
	Days to anthesis, Days to maturity	DH population	6
	Leaf length, Shoot length	RIL population	7
	Plant height, Shoot biomass, Grain yield, Harvest index, Maxium root length, Total root biomass, Root biomass up to 30 cm	RIL population	8
	TGW (locus 4B-b)	RIL population	9
	Yield (MQTL1.4, <400 kb)	Meta-QTL	10
	TGW, Yield, Plant height (locus 4B-b)	RIL population	11
	Yield, Plant height (locus 4B-b)	RIL population	12
Durum wheat	Canopy temperature depression, Water index, Quantum yield	RIL population	13
	MQTL17, etc	Meta-QTL	14
	Plant height	GWAS	15
	Plant height, TGW, Stress tolerance	GWAS	16
	Drought index	GWAS	17
	Root related traits, Yellow pigment content	Meta-QTL	18

Supplementary Figure 3. The drought tolerant QTL *qDSI-4B.1* in the short arm of chromosome 4B of hexaploid bread wheat and tetraploid durum wheat reported in the past fifteen years. a: The diagram of *qDSI-4B.1* and adjacent loci and genes associated with yield and plant height. **b**: The references that reported locus *qDSI-4B.1*.

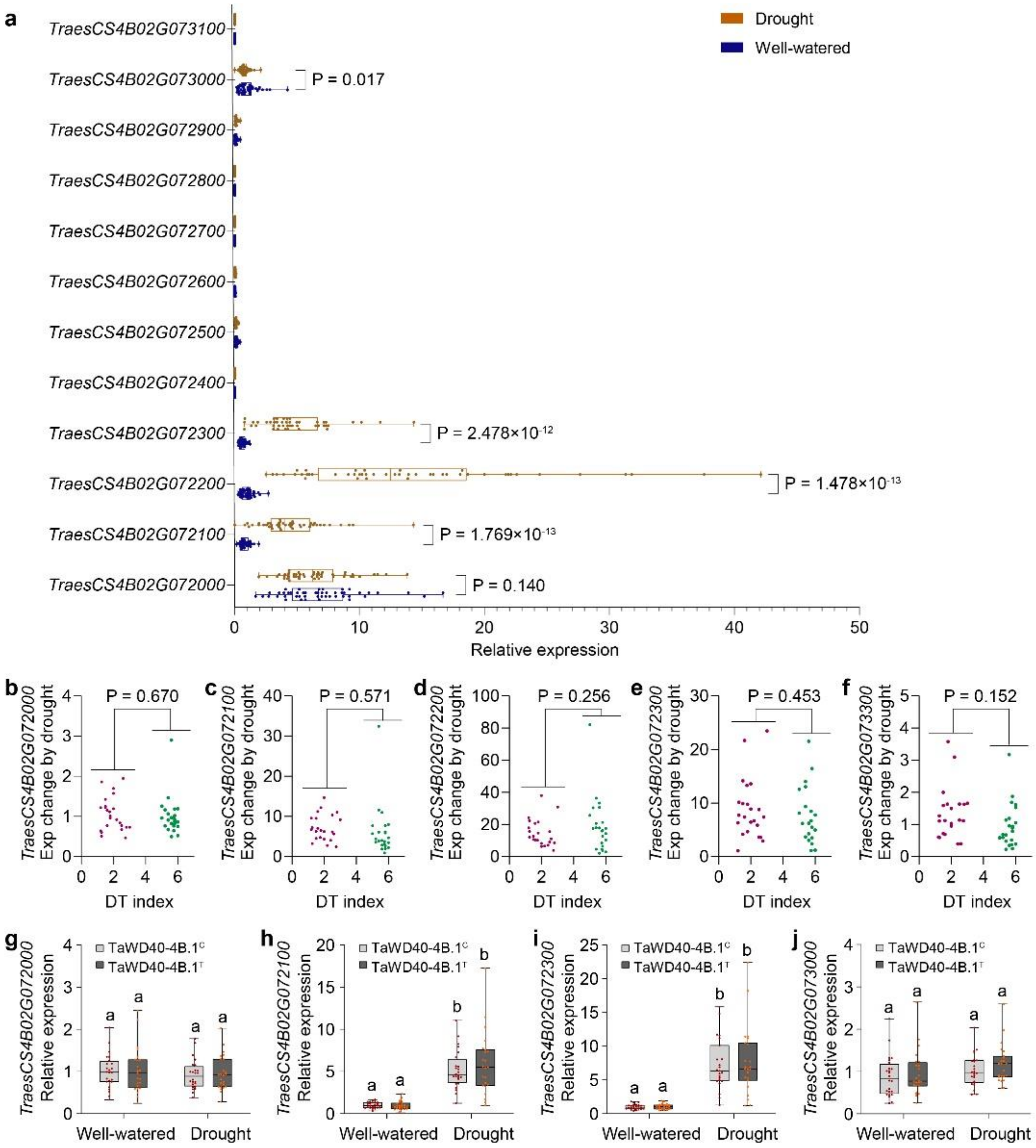

Supplementary Figure 4. The expression of twelve genes in the LD block of chromosome 4B. a: The relative expression levels of 48 selected accessions under well-watered and water-withheld conditions. The difference between well-watered and water-withheld conditions was calculated with a paired two-sided two-sample *t*-test (n = 48 wheat accessions; $P = 0.140$, 1.77×10^{-13}, 1.48×10^{-13}, 2.48×10^{-12}, 0.154, 2.61×10^{-4}, 0.018, 0.529 and 0.017 from TraesCS4B02G072000 to TraesCS4B02G073000 expect for TraesCS4B02G072700, TraesCS4B02G072800 and TraesCS4B02G073100). **b**-**f**: The comparison of expression alteration folds by drought between accessions with low and high DT indices. The difference was calculated via the independent two-sample two-sided *t*-test (n = 24 wheat accessions; $P = 0.670$, 0.571, 0.256, 0.453 and 0.152 respectively). **g**-**j**: The relative expression of genes in the accessions harboring *TaWD40-4B.1*^{C} and *TaWD40-4B.1*^{T} under well-watered and water-withheld conditions (n = 24 wheat accessions; $P = 0.840$, 4.19×10^{-14}, 4.87×10^{-13} and 0.327 respectively). The significance of the difference is calculated with a one-way ANOVA analysis – Tukey comparison and the columns labeled without the same alphabet are significantly different ($P < 0.05$, two-sided). In **a**, **g**-**j**: box indicates the range from lower to upper quartiles, and bar ranges the minimum to maximum observations. Source data are provided as a Source Data file.

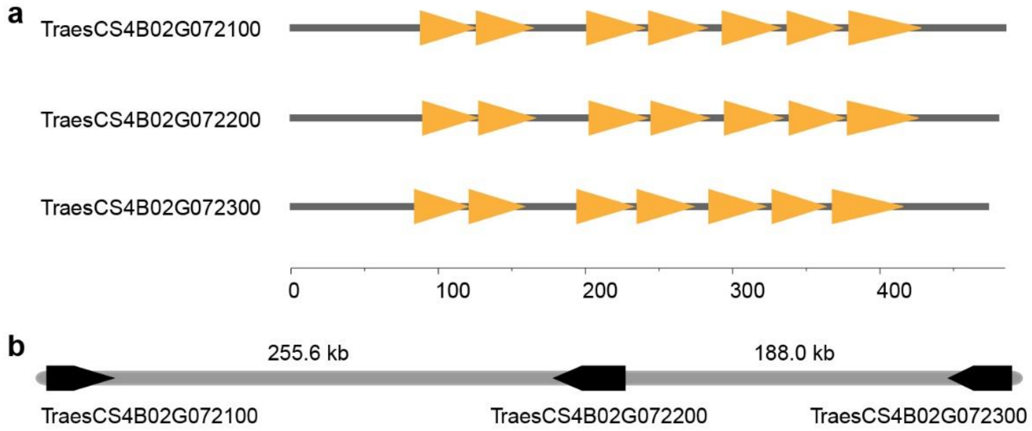

Supplementary Figure 5. The domain analysis of TaWD40-4B.1 and its paralogues. **a**: The WD40 domain analysis. **b**: The position of *TaWD40-4B.1* and its paralogues on chromosome 4B.

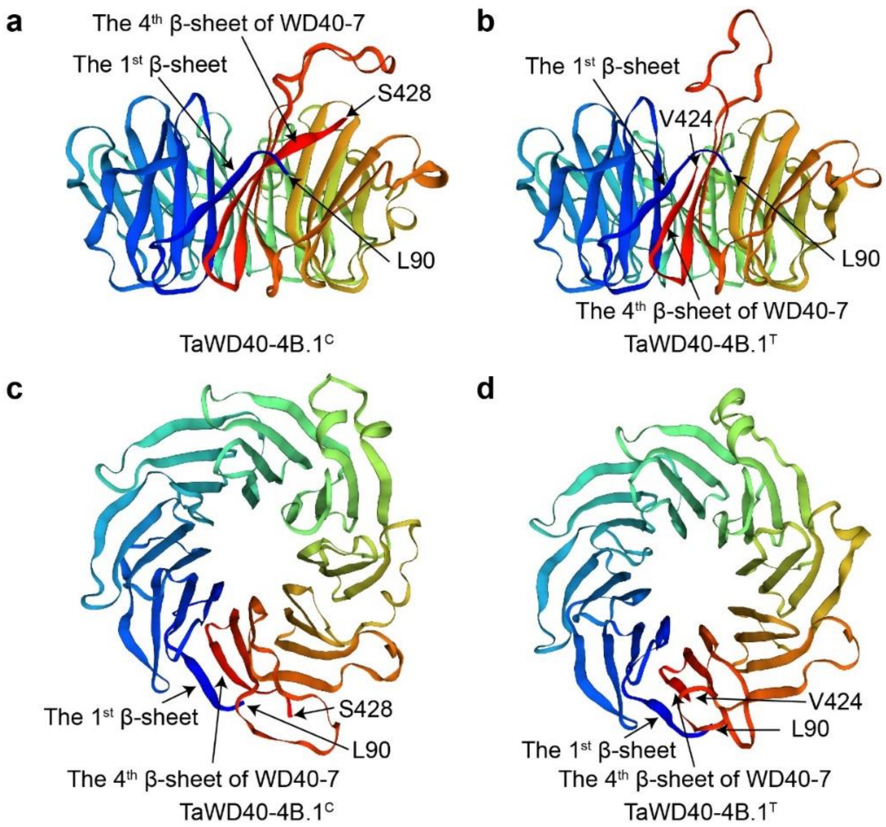

Supplementary Figure 6. The 3D structure of TaWD40-4B.1 predicted with SWISS-MODEL (https://swissmodel.expasy.org/). a, **c**: The structure of TaWD40-4B.1^{C}. **b**, **d**: The structure of TaWD40-4B.1^{T}.

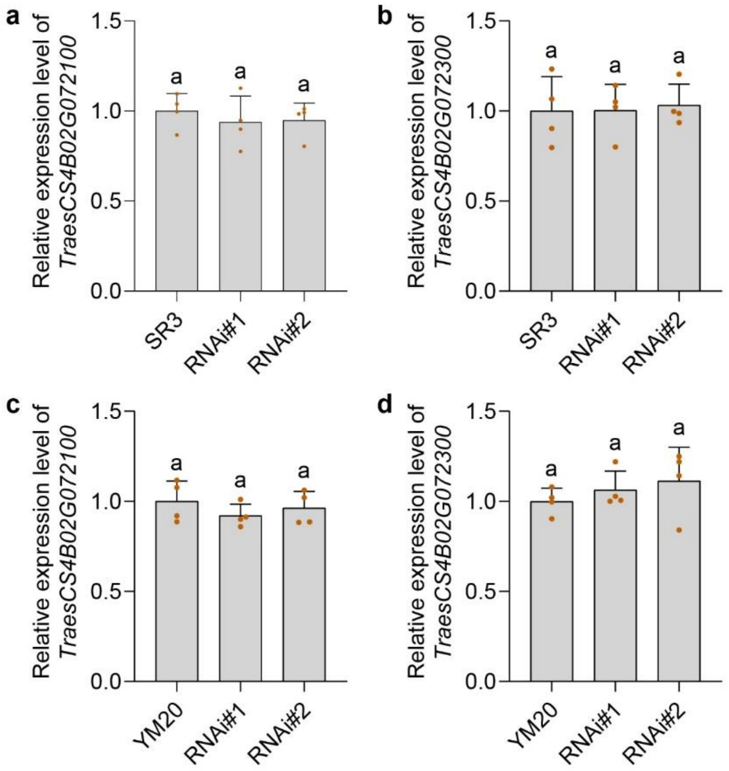

Supplementary Figure 7. The expression of two paralogues of *TaWD40-4B.1* in the *TaWD40-4B.1* RNAi lines. Data are shown as mean and standard deviation. The significance of the difference is calculated with a one-way ANOVA analysis – Tukey comparison and the columns labeled without the same alphabet are significantly different ($P < 0.05$, two-sided) (n = 4 biologically independent samples; $P = 0.728$, 0.952, 0.504 and 0.501 respectively in panels a-d). SR3 and YM20: two cultivars carrying *TaWD40-4B.1*^{C} and *TaWD40-4B.1*^{T} respectively; RNAi: *TaWD40-4B.1*^{C} / *TaWD40-4B.1*^{T} RNAi lines. Source data are provided as a Source Data file.

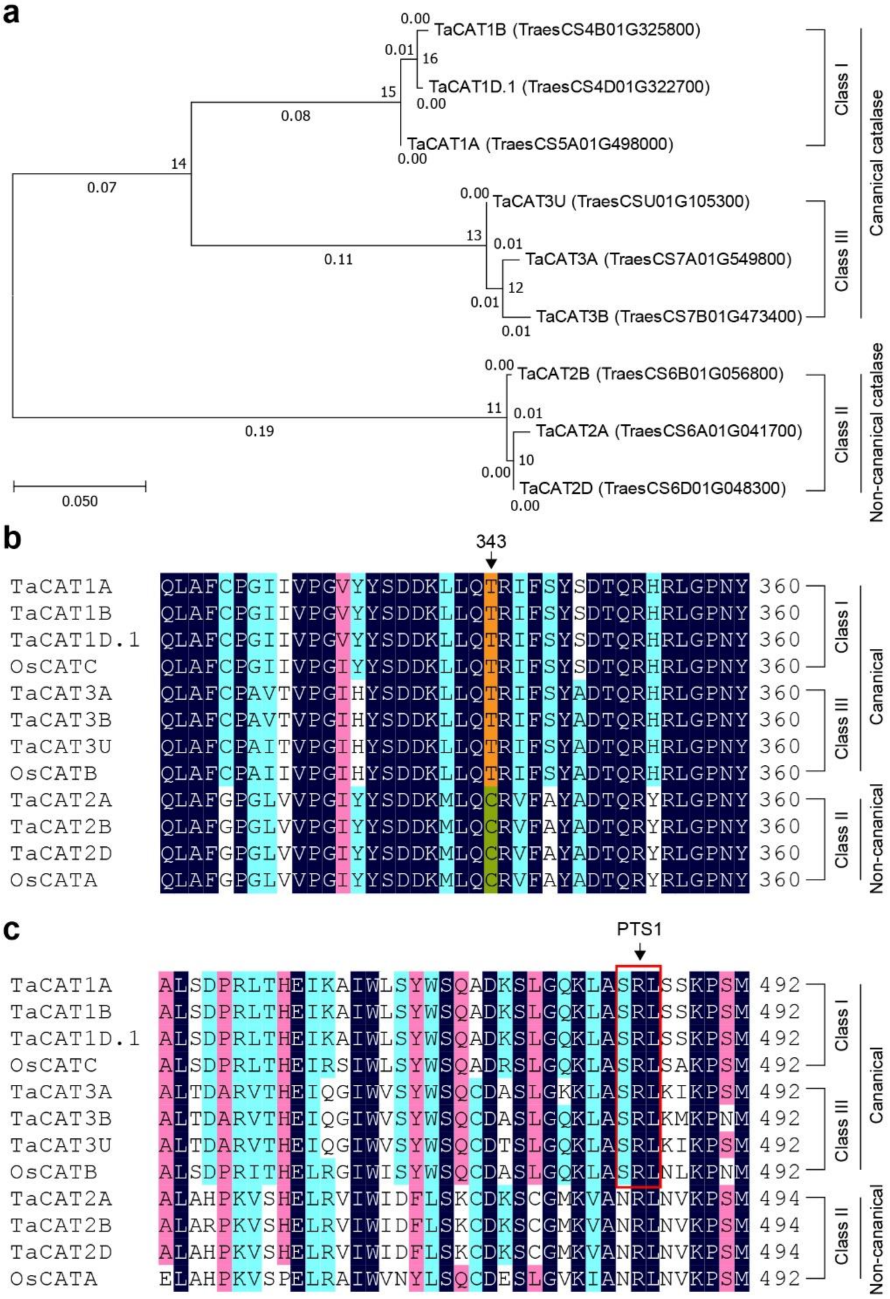

Supplementary Figure 8. The phylogenetic and sequence analysis of TaCATs.

a: The phylogenetic tree of TaCATs with the Neighbor-Joining method. **b**: The sequence alignment of wheat and rice catalase fragments containing the amino acid residue 343 that characterizes canonical and non-canonical catalase. **c**: The sequence alignment of wheat and rice catalase fragments containing the peroxisome targeting sequence at C-terminus.

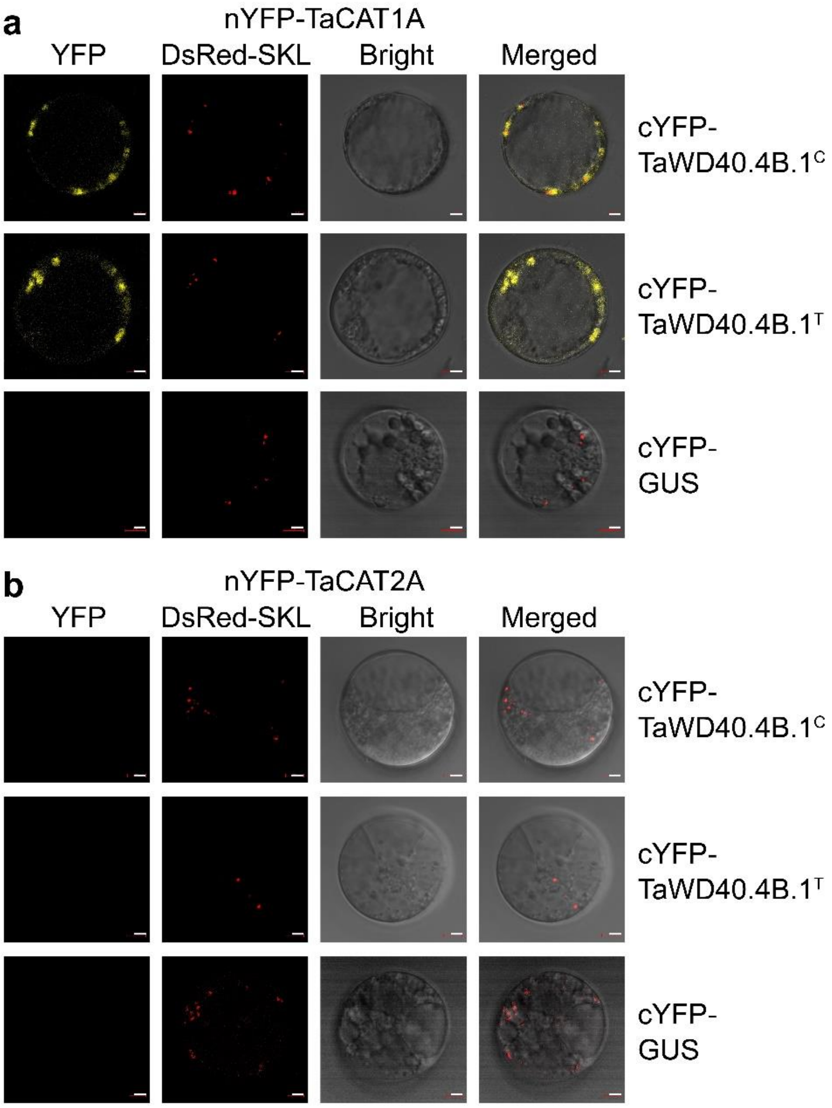

Supplementary Figure 9. The interaction of TaWD40-4B.1^{C} / TaWD40-4B.1^{T} and TaCAT1A / TaCAT2A. a, **b**: The bimolecular fluorescence complementation assay confirms the interaction between TaWD40-4B.1^{C} / TaWD40-4B.1^{T} and TaCAT1A / 2A. DsRed-SKL is the DsRed fused with peroxisome targeting sequence SKL serving as the peroxisome marker. Bar = 5 μm.

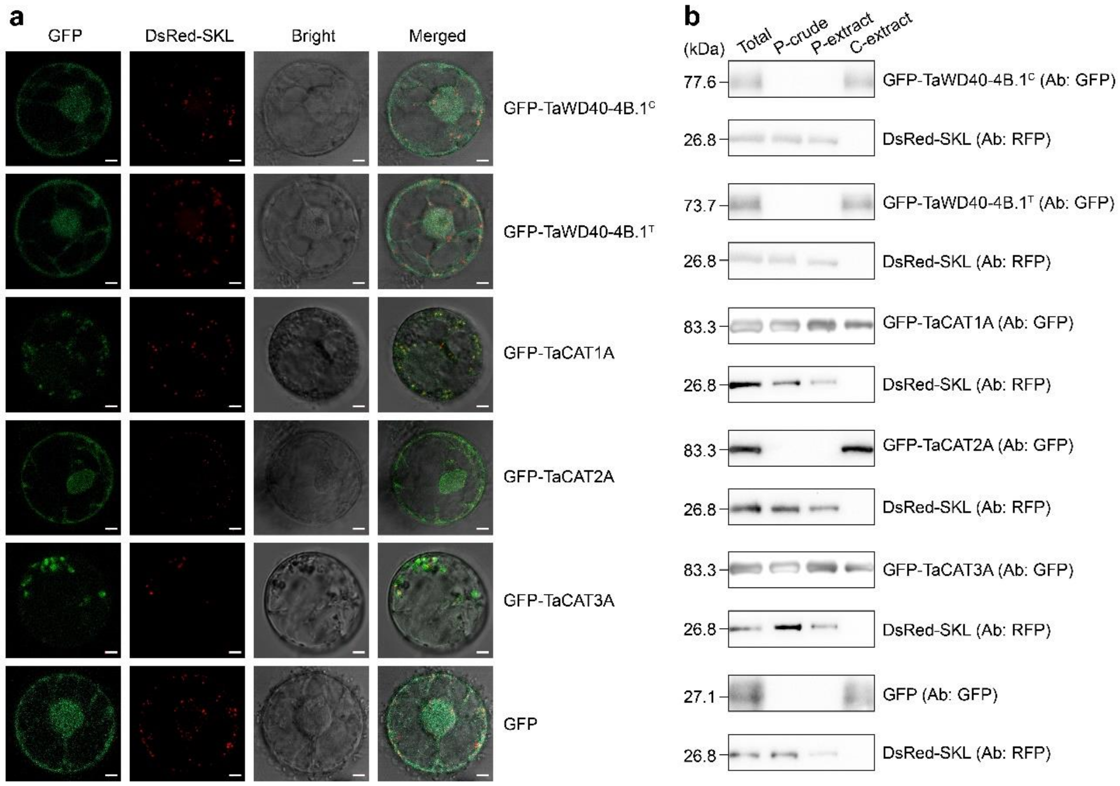

Supplementary Figure 10. The subcellular localization of TaWD40-4B.1^{C} / TaWD40-4B.1^{T} and TaCATs. a: Transient co-expression of GFP fused TaWD40-4B.1^{C}, TaWD40-4B.1^{T} or TaCATs with the peroxisome marker DsRed-SKL. **b**: The western blotting assay using the total, peroxisomal and cytosol proteins extracted from the protoplasts. Total, total proteins; P-crude: crude peroxisomal extracts; P-extract: peroxisomal extracts; C-extract: cytosol proteins. Source data are provided as a Source Data file.

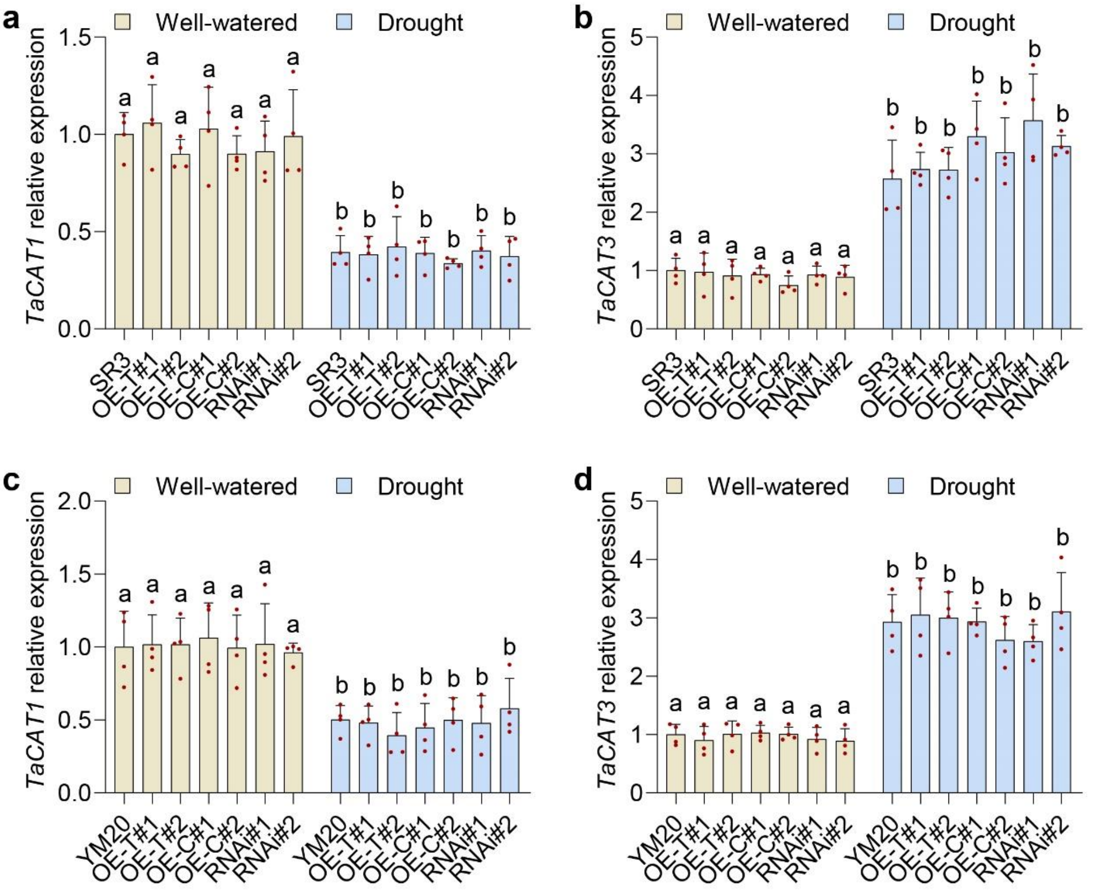

Supplementary Figure 11. *TaWD40-4B.1*^{C} and *TaWD40-4B.1*^{T} do not affect the expression of catalase genes. Data are shown as mean and standard deviation. The significance of the difference is calculated with a one-way ANOVA analysis – Tukey comparison and the columns labeled without the same alphabet are significantly different ($P < 0.05$, two-sided) (n = 4 biologically independent samples; $P = 4.15 \times 10^{-14}$, 7.32×10^{-17}, 4.01×10^{-8} and 1.87×10^{-18} respectively in panels a-d). Source data are provided as a Source Data file.

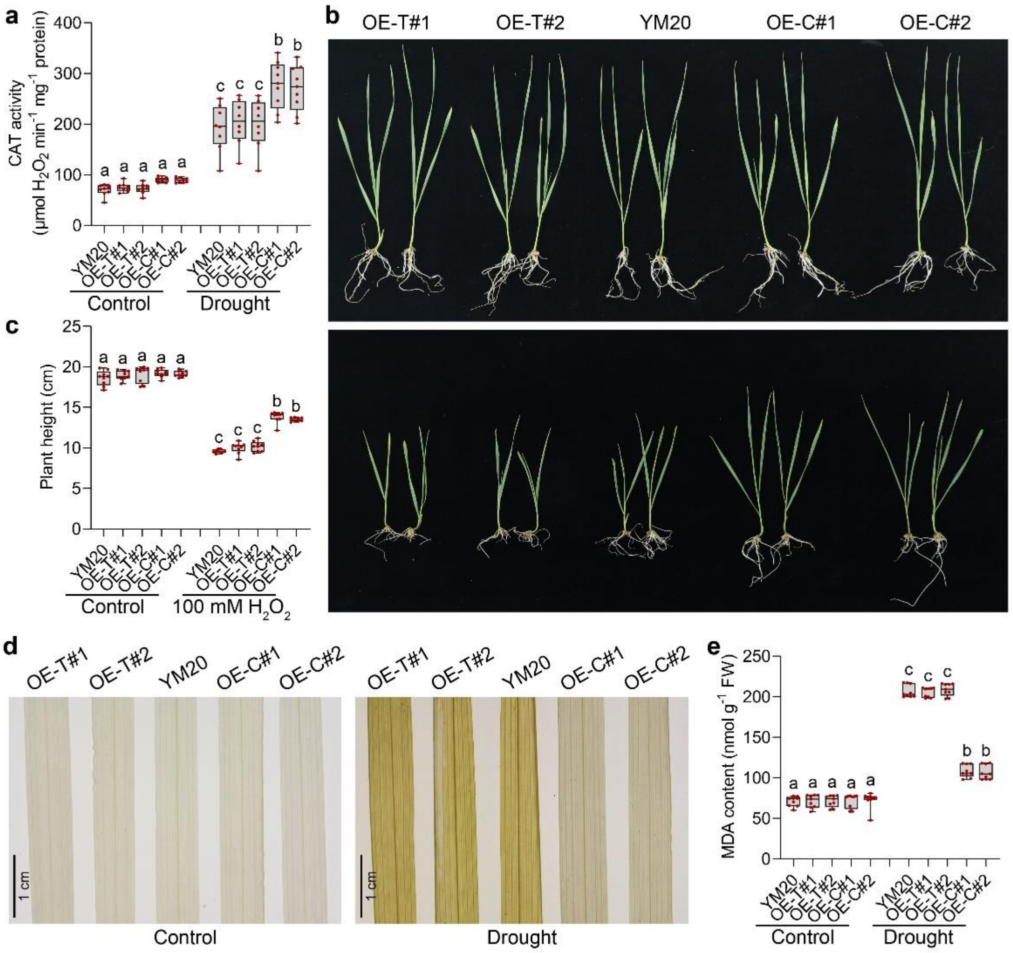

Supplementary Figure 12. *TaWD40-4B.1*^{C} but not *TaWD40-4B.1*^{T} enhances tolerance to oxidative stress and reduces H_2O_2 level under drought in YM20. a: The catalase activities in leaves of YM20 and its transgenic lines under the well-watered and water-withheld conditions (n = 9 biologically independent samples; $P = 1.19 \times 10^{-30}$). **b**: The assessment of the oxidative stress tolerance of SR3 and its transgenic lines. **c**: The statistical result of plant height in panel **b** (n = 9 biologically independent samples; $P = 4.62 \times 10^{-63}$). **d**: the H_2O_2 levels revealed by DAB staining indicates in the leaves of wheat seedlings under the well-watered and water-withheld conditions. **e**: The MDA contents in the leaves of wheat seedlings under the well-watered and water-withheld conditions (n = 9 biologically independent samples; $P = 8.03 \times 10^{-69}$). In **a**, **c** and **e**, box indicates the range from lower to upper quartiles, and bar ranges the minimum to maximum observations; the significance of the difference is calculated with a one-way ANOVA analysis – Tukey comparison and the columns labeled without the same alphabet are significantly different ($P < 0.05$, two-sided). YM20: the cultivar carrying *TaWD40-4B.1*^{T}; OE-T: *TaWD40-4B.1*^{T} overexpression lines; OE-C: *TaWD40-4B.1*^{C} overexpression lines. Source data are provided as a Source Data file.

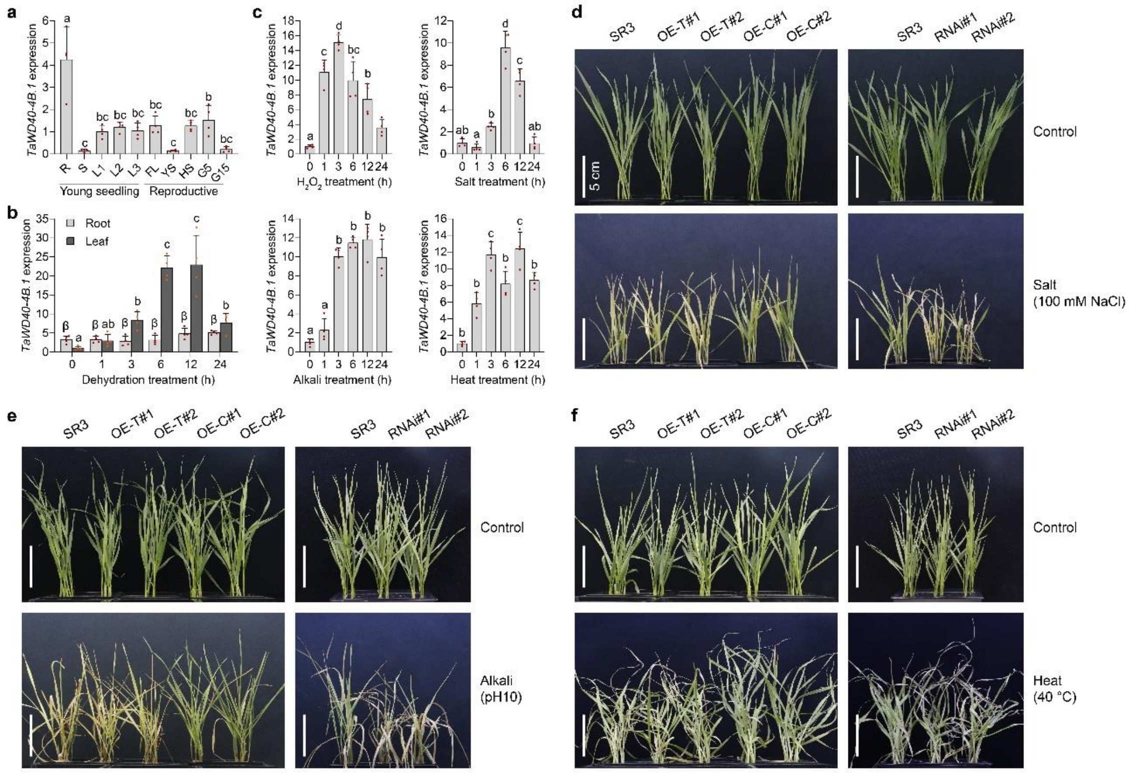

Supplementary Figure 13. ***TaWD40-4B.1***^{C} **enhances the tolerance to salt, alkali and heat stress. a**: The expression of *TaWD40-4B.1*^{C} in different tissues at the three-leaf and reproductive stages (n = 4 biologically independent samples; $P = 6.75 \times 10^{-10}$). R, S, L1, L2 and L3: roots, stems, the first, second and third leaves of SR3 seedlings at three-leaf stage; FL, flag leaves at heading stage; YS, young spikes at early booting stage; HS, spikes at heading stage; G5 and G15, the grains of 5 and 15 days of post anthesis, respectively. **b**: The transcriptional profiles of *TaWD40-4B.1* in the leaves and roots of SR3 seedlings under dehydration (n = 4 biologically independent samples; $P = 0.033$ in roots and 1.31×10^{-7} in leaves). **c**: The expression of *TaWD40-4B.1*^{C} in the leaves of SR3 seedlings treated with H_2O_2, salt, alkali and heat (n =4 biologically independent samples; $P = 4.07 \times 10^{-9}$, 7.57×10^{-12}, 1.17×10^{-10} and 7.08×10^{-9} respectively). 0~24: the time of treatment. **d-f**: The phenotypes under salt (**d**), alkali (**e**) and heat (**f**) stresses. Bar: 5 cm. In **a**-**c**, data are shown as mean and standard deviation; the significance of the difference is calculated with a one-way ANOVA analysis – Tukey comparison and the columns labeled without the same alphabet are significantly different ($P < 0.05$, two-sided). SR3: the cultivar carrying *TaWD40-4B.1*^{C}; OE-T: *TaWD40-4B.1*^{T} overexpression lines; OE-C: *TaWD40-4B.1*^{C} overexpression lines; RNAi: *TaWD40-4B.1*^{C} RNAi lines. Source data are provided as a Source Data file.

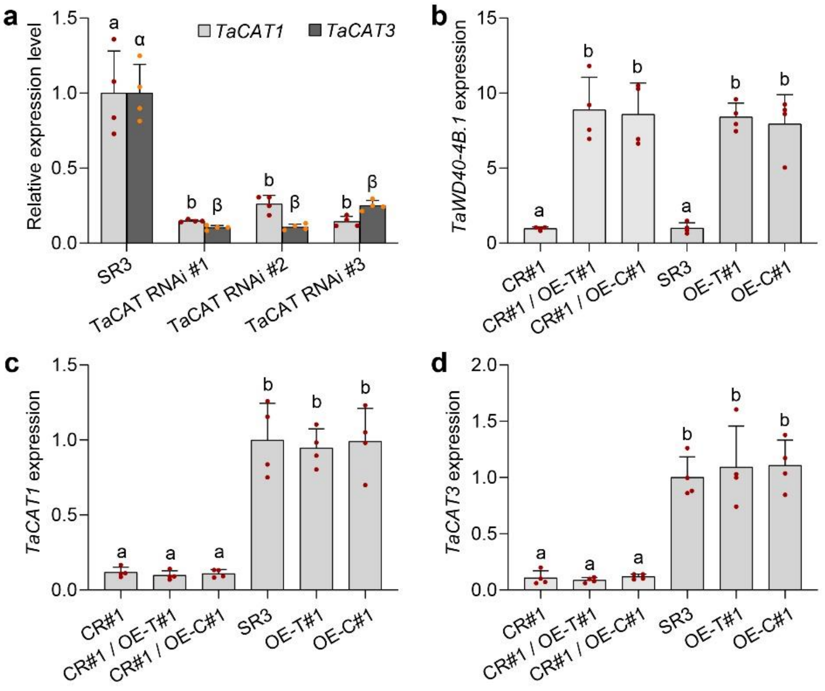

Supplementary Figure 14. The expression of catalase genes and *TaWD40-4B.1* in the *TaCAT* RNAi lines and *TaCAT* RNAi / *TaWD40-4B.1* overexpression lines. a: The expression of canonical catalase genes *TaCAT1* and *TaCAT3* in the *TaCAT* RNAi lines (n = 4 biologically independent samples; $P = 4.70 \times 10^{-6}$ for *TaCAT1* and 4.01×10^{-8} *TaCAT3*). **b-d**: The expression of *TaWD40-4B.1*, *TaCAT1* and *TaCAT3* in the cross lines (n = 4 biologically independent samples; $P = 1.27 \times 10^{-7}$, 2.27×10^{-9} and 3.71×10^{-8} in panels b-d). Data are shown as mean and standard deviation. The significance of the difference is calculated with a one-way ANOVA analysis – Tukey comparison and the columns labeled without the same alphabet are significantly different ($P < 0.05$, two-sided). RNAi#1-3: three *TaCAT* RNAi lines of SR3 carrying *TaWD40-4B.1^C*; OE-C#1: *TaWD40-4B.1^C* overexpression line; OE-T#1: *TaWD40-4B.1^T* overexpression line; CR#1 / OE-T#1: *TaCAT* RNAi / *TaWD40-4B.1^T* overexpression cross line; CR#1 / OE-C#1: *TaCAT* RNAi / *TaWD40-4B.1^C* overexpression cross line. Source data are provided as a Source Data file.

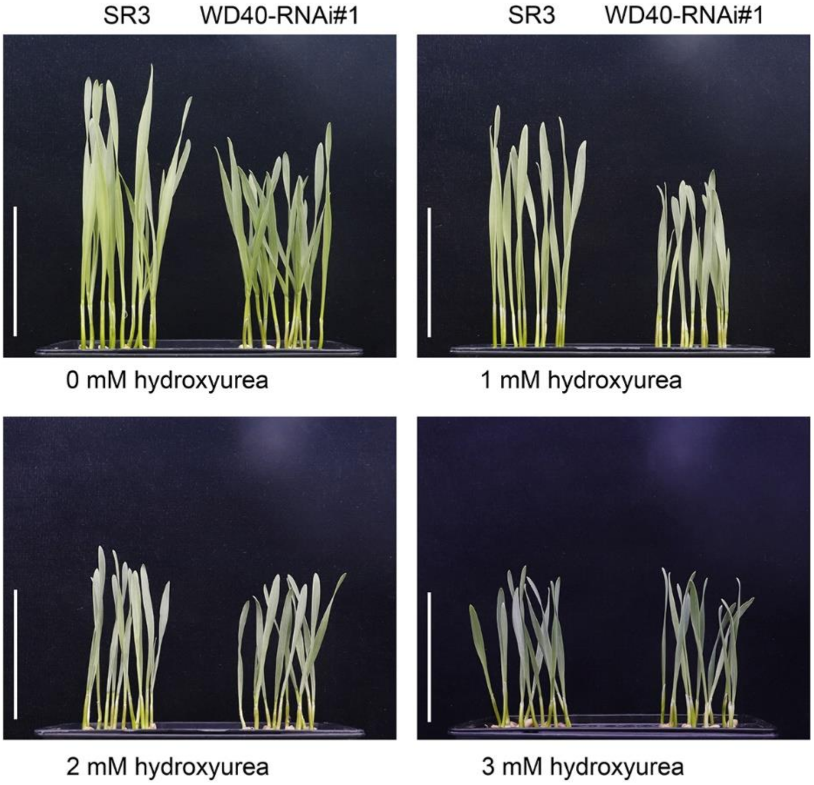

Supplementary Figure 15. The effect of on the growth of wheat seedlings under high light intensity. Bar = 5 cm. SR3: the cultivar carrying *TaWD40-4B.1*^{C}; WD40-RNAi#1: *TaWD40-4B.1*^{C} RNAi line.

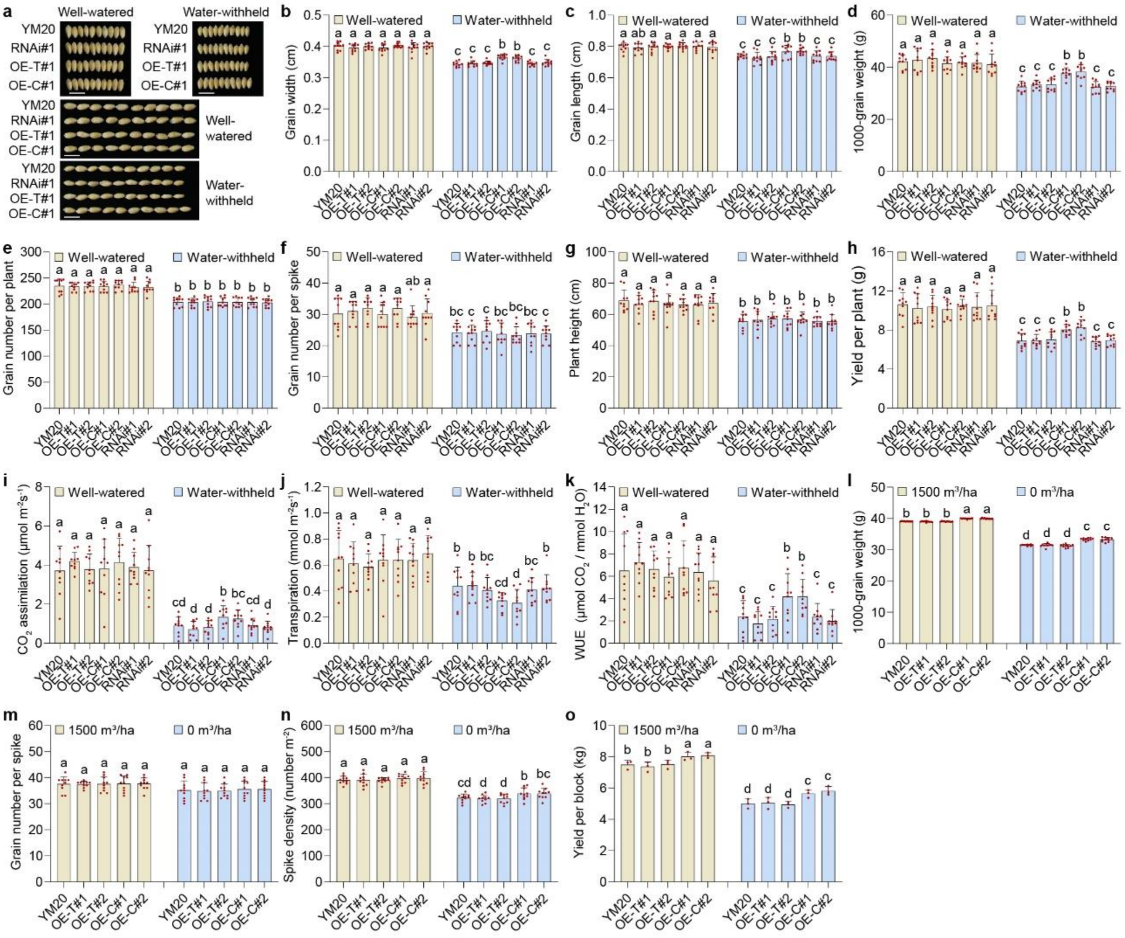

Supplementary Figure 16. ***TaWD40-4B.1^{C}*** **enhances grain yields of YM20 in water-withheld condition.** **a**: The grain sizes of SR3 and its transgenic lines under the well-watered and water-withheld conditions. **b**, **c**: The statistical comparison of grain width (b) and length (c) of panel a (n = 10 biologically independent samples; $P = 1.41 \times 10^{-48}$ and 4.93×10^{-15} respectively). **d**: The 1000-grain weights under the well-watered and water-withheld conditions (n = 10 biologically independent samples; $P = 1.16 \times 10^{-28}$). **e-g**: The grain number per plant (e), grain number per spike (f), and plant height (g) under the well-watered and water-withheld conditions (n = 10 biologically independent samples; $P = 3.09 \times 10^{-31}$, 1.34×10^{-14} and 1.15×10^{-14} respectively in panels e-g). **h**: The grain yield of plants under the well-watered and water-withheld conditions (n = 10 biologically independent samples; $P = 2.36 \times 10^{-30}$). **i-k**: The CO_2 assimilation (i), transpiration rate (j) and water use efficiency (k) under the well-watered and water-withheld conditions (n = 10 biologically independent samples; $P = 6.65 \times 10^{-35}$, 2.11×10^{-13} and 1.12×10^{-17} respectively in panels i-k). **l-o**: 1000-grain weights (l), spike numbers per spike (m), spike densities (n) and grain yields (o) in the field trail (n = 10 for panels l-n and 3 for panel o (biologically independent samples); $P = 1.15 \times 10^{-89}$, 0.051, 2.37×10^{-26} and 4.92×10^{-13} respectively in panels l-o). In **b-o**, data are shown as mean and standard deviation; the significance of the difference is calculated with a one-way ANOVA analysis – Tukey comparison and the columns labeled without the same alphabet are significantly different ($P < 0.05$, two-sided). YM20: the cultivar carrying *TaWD40-4B.1^{T}*; OE-T: *TaWD40-4B.1^{T}* overexpression lines; OE-C: *TaWD40-4B.1^{C}* overexpression lines; RNAi: *TaWD40-4B.1^{T}* RNAi lines. Source data are provided as a Source Data file.

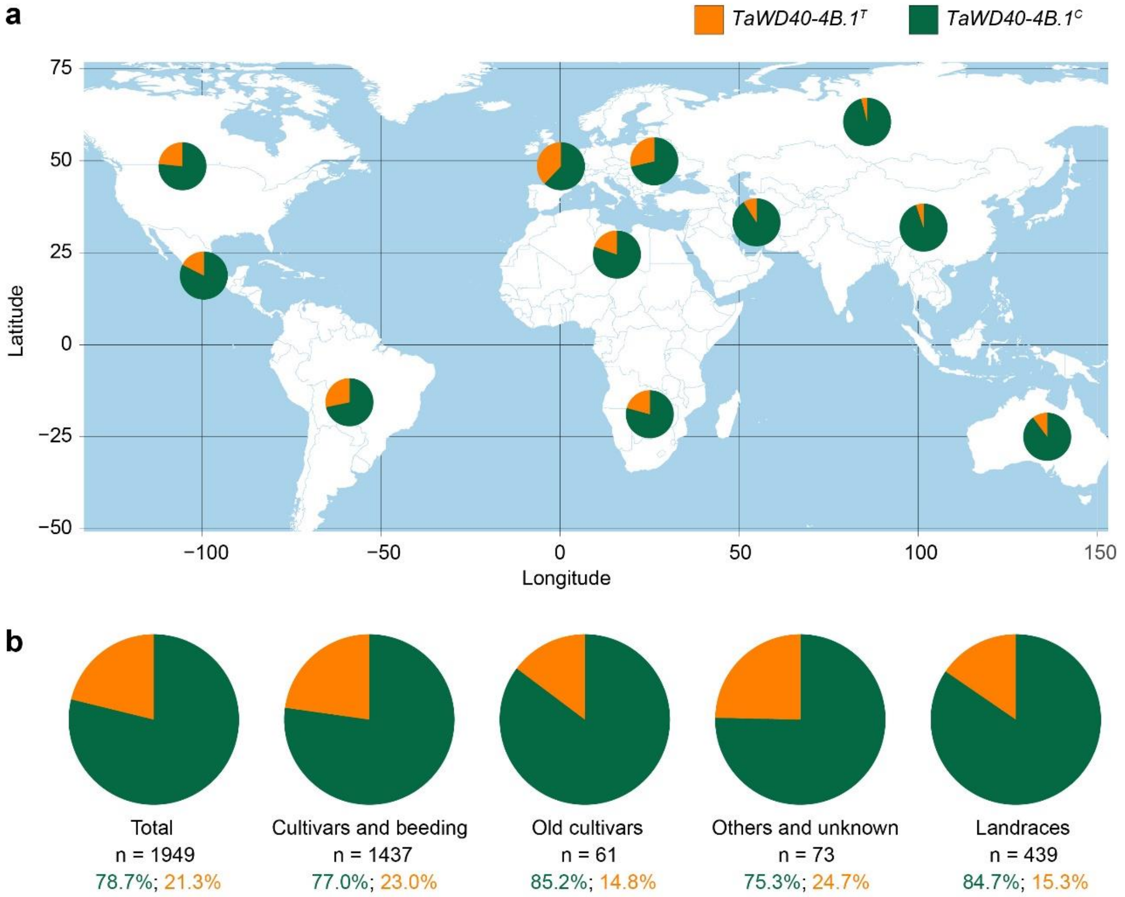

Supplementary Figure 17. The distribution and proportion of accessions carrying *TaWD40-4B.1^C* and *TaWD40-4B.1^T* around the world. a: The distribution of *TaWD40-4B.1^C* and *TaWD40-4B.1^T*. **b**. The proportions of *TaWD40-4B.1^C* and *TaWD40-4B.1^T* in 1861 wheat accessions.

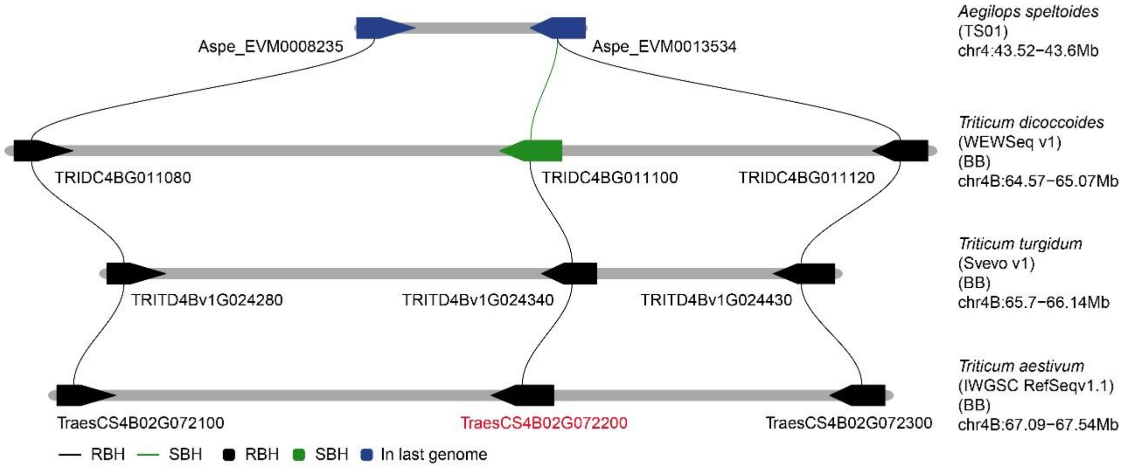

Supplementary Figure 18. The collinearity analysis of *TaWD40-4B.1* and its paralogues and homologues. The genes of hexaploid and tetraploid wheat and diploid progenitor close species *Aegilops speltoides* were used for analysis.

Supplementary references

1 Nouraei, S., Mia, M. S., Liu, H., Turner, N. C. & Yan, G. Transcriptome analyses of near isogenic lines reveal putative drought tolerance controlling genes in wheat. *Front Plant Sci* **13**, 857829 (2022).

2 Siddiqui, M. N. *et al.* New drought-adaptive loci underlying candidate genes on wheat chromosome 4B with improved photosynthesis and yield responses. *Physiol Plant* **173**, 2166–2180 (2021).

3 Nagy, É. *et al.* Detection of drought tolerance-related QTL in the Plainsman V./Cappelle Desprez doubled haploid wheat population. *Cereal Res Communs* **50**, 689–698 (2022).

4 Liu, H. *et al.* Major genomic regions responsible for wheat yield and its components as revealed by meta-QTL and genotype–phenotype association analyses. *Planta* **252**, 65 (2020).

5 Puttamadanayaka, S. *et al.* Mapping genomic regions of moisture deficit stress tolerance using backcross inbred lines in wheat (*Triticum aestivum* L.). *Sci Rep* **10**, 21646 (2020).

6 Gahlaut, V. *et al.* QTL mapping for nine drought-responsive agronomic traits in bread wheat under irrigated and rain-fed environments. *PLoS One* **12**, e0182857 (2017).

7 Zhang, H. *et al.* Conditional and unconditional QTL mapping of drought-tolerance-related traits of wheat seedling using two related RIL populations. *J Genet* **92**, 213-231 (2013).

8 Kadam, S. *et al.* Genomic associations for drought tolerance on the short arm of wheat chromosome 4B. *Funct Integr Genomics* **12**, 447-464 (2012).

9 Nezhad, K. Z. *et al.* QTL analysis for thousand-grain weight under terminal drought stress in bread wheat (*Triticum aestivum* L.). *Euphytica* **186**, 127-138 (2012).

10 Swamy, B. M., Vikram, P., Dixit, S., Ahmed, H. & Kumar, A. Meta-analysis of grain yield QTL identified during agricultural drought in grasses showed consensus. *BMC Genomics* **12**, 319 (2011).

11 Pinto, R. S. *et al.* Heat and drought adaptive QTL in a wheat population designed to minimize confounding agronomic effects. *Theor Appl Genet* **121**, 1001-1021 (2010).

12 Mathews, K. L. *et al.* Multi-environment QTL mixed models for drought stress adaptation in wheat. *Theor Appl Genet* **117**, 1077-1091 (2008).

13 Diab, A. A. *et al.* Drought - inducible genes and differentially expressed sequence tags associated with components of drought tolerance in durum wheat. *Sci Res Essay* **3**, 9-26 (2008).

14 Iannucci, A. *et al.* Mapping QTL for root and shoot morphological yraits in a durum wheat × *T. dicoccum* segregating population at seedling stage. *Int J Genomics* **2017**, 6876393 (2017).

15 Soriano, J. M., Malosetti, M., Roselló, M., Sorrells, M. E. & Royo, C. Dissecting the old Mediterranean durum wheat genetic architecture for phenology, biomass and yield formation by association mapping and QTL meta-analysis. *PLoS One*

12, e0178290 (2017).
16 Sukumaran, S., Reynolds, M. P. & Sansaloni, C. Genome-wide association analyses identify QTL hotspots for yield and component traits in durum wheat grown under yield potential, drought, and heat stress environments. *Front Plant Sci* **9** (2018).
17 Wang, S. *et al.* A Genome-wide association study of highly heritable agronomic traits in durum wheat. *Front Plant Sci* **10**, 919 (2019).
18 Soriano, J. M., Colasuonno, P., Marcotuli, I. & Gadaleta, A. Meta-QTL analysis and identification of candidate genes for quality, abiotic and biotic stress in durum wheat. *Sci Rep* **11**, 11877 (2021).
