# Supplementary material for: Allelic variation of TaWD40-4B.1 contributes to drought tolerance by modulating catalase activity in wheat
Source: Nat Commun. 2023 Mar 2;14:1200. doi: 10.1038/s41467-023-36901-6 (PMC9981739; doi:10.1038/s41467-023-36901-6)
Supplement: Supplementary file 3 — Description of Additional Supplementary Files [file 41467_2023_36901_MOESM3_ESM.pdf]

Description of Additional Supplementary Information

Supplementary Data 1. The 198 wheat accessions and their drought tolerance phenotype (DT index) used for GWAS.

Supplementary Data 2. The summary of provenance, breeding status, and population structure inferred by the admixture model in the ADMIXTURE software package of the wheat diversity panel.

Supplementary Data 3. Information of significant SNP-trait associations in locus qDT4B.

Supplementary Data 4. Annotated genes within the region of identified qDT4B locus.

Supplementary Data 5. The accessions used for *TaWD40-4B.1* expression and catalase activity measurement.

Supplementary Data 6. SNPs in Ta*WD40-4B.1* in the population.

Supplementary Data 7. The domain prediction of the WD40 domain with SMART (https://smart.embl-heidelberg.de/).

Supplementary Data 8. Distribution of *TaWD40-4B.1* alleles in the population.

Supplementary Data 9. The proportions of *TaWD40-4B.1* alleles and annual rainfall in China.

Supplementary Data 10. The distribution of *TaWD40-4B.1* alleles around the world without China.

Supplementary Data 11. The proportions of *TaWD40-4B.1* alleles around the world.

Supplementary Data 12. The haplotype of *TaWD40-4B.1*'s homologues in tetraploid emmer and durum wheat.

Supplementary Data 13. Primers used for gene expression and vector construction.
