# Supplementary material for: Allelic variation of TaWD40-4B.1 contributes to drought tolerance by modulating catalase activity in wheat
Source: Nat Commun. 2023 Mar 2;14:1200. doi: 10.1038/s41467-023-36901-6 (PMC9981739; doi:10.1038/s41467-023-36901-6)
Supplement: Supplementary file 5 — Reporting Summary [file 41467_2023_36901_MOESM5_ESM.pdf]

nature portfolio

Corresponding author(s): *Double-blind peer review submissions: write DBPR and your manuscript number here instead of author names.*

Last updated by author(s): *YYYY-MM-DD*

Reporting Summary

Nature Portfolio wishes to improve the reproducibility of the work that we publish. This form provides structure for consistency and transparency in reporting. For further information on Nature Portfolio policies, see our Editorial Policies and the Editorial Policy Checklist.

Statistics

For all statistical analyses, confirm that the following items are present in the figure legend, table legend, main text, or Methods section.

n/a	Confirmed	
☐	☒	The exact sample size (*n*) for each experimental group/condition, given as a discrete number and unit of measurement
☐	☒	A statement on whether measurements were taken from distinct samples or whether the same sample was measured repeatedly
☐	☒	The statistical test(s) used AND whether they are one- or two-sided<br>*Only common tests should be described solely by name; describe more complex techniques in the Methods section.*
☒	☐	A description of all covariates tested
☐	☒	A description of any assumptions or corrections, such as tests of normality and adjustment for multiple comparisons
☐	☒	A full description of the statistical parameters including central tendency (e.g. means) or other basic estimates (e.g. regression coefficient) AND variation (e.g. standard deviation) or associated estimates of uncertainty (e.g. confidence intervals)
☐	☒	For null hypothesis testing, the test statistic (e.g. *F*, *t*, *r*) with confidence intervals, effect sizes, degrees of freedom and *P* value noted<br>*Give P values as exact values whenever suitable.*
☒	☐	For Bayesian analysis, information on the choice of priors and Markov chain Monte Carlo settings
☒	☐	For hierarchical and complex designs, identification of the appropriate level for tests and full reporting of outcomes
☐	☒	Estimates of effect sizes (e.g. Cohen's *d*, Pearson's *r*), indicating how they were calculated

Our web collection on statistics for biologists contains articles on many of the points above.

Software and code

Policy information about availability of computer code

Data collection	The SNP data of 1312 wheat varieties was referred to SnpHub(http://wheat.cau.edu.cn/Wheat_SnpHub_Portal/).
Data analysis	SPSS was used for statistical analysis of data; GraphPad Prism 9 were used to analyze the data; Adobe Illustrator was used for Figure assembly; Image J (vl.8.0) was used to analyze the plant height; PLINK (v1.9) was used for QC of SNP; ADMIXTURE (v1.3.0) was used to population structure investigation. GWAS was performed using the GAPIT R package; LD and haplotype blocks were constructed using the LDBlockShow (v1.40).

For manuscripts utilizing custom algorithms or software that are central to the research but not yet described in published literature, software must be made available to editors and reviewers. We strongly encourage code deposition in a community repository (e.g. GitHub). See the Nature Portfolio guidelines for submitting code & software for further information.

Data

Policy information about availability of data

All manuscripts must include a data availability statement. This statement should provide the following information, where applicable:

- Accession codes, unique identifiers, or web links for publicly available datasets
- A description of any restrictions on data availability
- For clinical datasets or third party data, please ensure that the statement adheres to our policy

We have no this kind of data.

Human research participants

Policy information about studies involving human research participants and Sex and Gender in Research.

Reporting on sex and gender	*Use the terms sex (biological attribute) and gender (shaped by social and cultural circumstances) carefully in order to avoid confusing both terms. Indicate if findings apply to only one sex or gender; describe whether sex and gender were considered in study design whether sex and/or gender was determined based on self-reporting or assigned and methods used. Provide in the source data disaggregated sex and gender data where this information has been collected, and consent has been obtained for sharing of individual-level data; provide overall numbers in this Reporting Summary. Please state if this information has not been collected. Report sex- and gender-based analyses where performed, justify reasons for lack of sex- and gender-based analysis.*
Population characteristics	*Describe the covariate-relevant population characteristics of the human research participants (e.g. age, genotypic information, past and current diagnosis and treatment categories). If you filled out the behavioural & social sciences study design questions and have nothing to add here, write "See above."*
Recruitment	*Describe how participants were recruited. Outline any potential self-selection bias or other biases that may be present and how these are likely to impact results.*
Ethics oversight	*Identify the organization(s) that approved the study protocol.*

Note that full information on the approval of the study protocol must also be provided in the manuscript.

Field-specific reporting

Please select the one below that is the best fit for your research. If you are not sure, read the appropriate sections before making your selection.

☒ Life sciences ☐ Behavioural & social sciences ☐ Ecological, evolutionary & environmental sciences

For a reference copy of the document with all sections, see nature.com/documents/nr-reporting-summary-flat.pdf

Life sciences study design

All studies must disclose on these points even when the disclosure is negative.

Sample size	The association-mapping panel composed of 198 wheat accessions was used for drought tolerance. The sample size were referring to a published paper titled "Genomic basis of geographical adaptation to soil nitrogen in rice" (doi: https://doi.org/10.1038/s41586-020-03091-w), which efficiently identified GWAS loci using only 110 samples/accessions.<br>For molecular and physiological experiments, at least three biologically independent samples were used to derive statistical analysis. The sample sizes are represented as scatters in the figures or described in the text.
Data exclusions	For the phenotyping of drought tolerance represented as DT index for GWAS, each accession had five replicates. However, the DT index that was significantly deviated from those of the other replicates was considered as the outlier and excluded from analysis.
Replication	For the phenotyping of drought tolerance for GWAS, each accession had five replicates.<br>All quantitative-analysis requiring experiments were performed in at least three independent biological replicates including technical replicates per experiment. Technical replicates were averaged within each biological replicate for further analysis. All figure legends show means and variability calculated from at least three independent experiments.
Randomization	For the phenotyping of drought tolerance in the experiments of GWAS with natural accessions, transgenic lines in lab as well as in field trails, all of experiments were conducted with completely random design.
Blinding	The experiments were not blinded, but repeated by multiple independent experiments with completely random design to avoid bias. A randomized complete block design with five biologically independent replications was adopted in phenotyping for GWAS. As a result, a credible genetic locus was identified and the causal gene was identified and validated in our study. Data analyses were blinded to plant selection.

Reporting for specific materials, systems and methods

We require information from authors about some types of materials, experimental systems and methods used in many studies. Here, indicate whether each material, system or method listed is relevant to your study. If you are not sure if a list item applies to your research, read the appropriate section before selecting a response.

Materials & experimental systems

n/a	Involved in the study	
[ ]	[x]	Antibodies
[x]	[ ]	Eukaryotic cell lines
[x]	[ ]	Palaeontology and archaeology
[x]	[ ]	Animals and other organisms
[x]	[ ]	Clinical data
[x]	[ ]	Dual use research of concern

Methods

n/a	Involved in the study	
[x]	[ ]	ChIP-seq
[x]	[ ]	Flow cytometry
[x]	[ ]	MRI-based neuroimaging

Antibodies

Antibodies used

Primary antibody; Mouse anti Myc-Tag mAb; Abclonal; AE010; dilution 1: 5000
Primary antibody; Mouse anti DDDDK-Tag mAb; Abclonal; AE005; dilution 1: 5000
Primary antibody; Mouse anti GFP-Tag mAb; Abclonal; AE012; dilution 1: 5000
Primary antibody; Mouse anti MBP-Tag mAb; Abclonal; AE016; dilution 1: 5000
Primary antibody; Mouse anti His-Tag mAb; Abclonal; AE003; dilution 1: 5000
rimary antibody; Mouse anti RFP-Tag mAb; Abclonal; AE020; dilution 1: 5000
CAT1 Antibody for Plant; Abmart; ZW014564; dilution 1: 5000
Secondary antibody; HRP Goat Anti-Mouse IgG (H+L); Abclonal; AS003; dilution 1: 5000

Validation

All antibodies are commercially available and widely used in the scientific community. The following antibodies were validated by the supplier:
Mouse anti Myc-Tag mAb:
https://abclonal.com.cn/catalog/AE010
Mouse anti DDDDK-Tag mAb:
https://abclonal.com.cn/catalog/AE005
Mouse anti GFP-Tag mAb:
https://abclonal.com.cn/catalog/AE012
Mouse anti MBP-Tag mAb
https://abclonal.com.cn/catalog/AE016
Mouse anti His-Tag mAb:
https://abclonal.com.cn/catalog/AE003
Mouse anti RFP-Tag mAb:
https://abclonal.com.cn/catalog/AE020
HRP Goat Anti-Mouse IgG (H+L)
https://abclonal.com.cn/catalog/AS003
